# A Very Complicated Inferior Myocardial Infarction: The Role of Multimodality Imaging Approach

**DOI:** 10.5935/abc.20160073

**Published:** 2016-05

**Authors:** Inês Cruz, Carlos Cotrim, Luís Lopes, Paula Fazendas, Hélder Pereira

**Affiliations:** Cardiology Department, Hospital Garcia de Orta, Almada - Portugal

**Keywords:** Myocardial Infarction, Ventricular Septal Rupture, Aneurysm, Myocardial Revascularization

**Figure 1:**
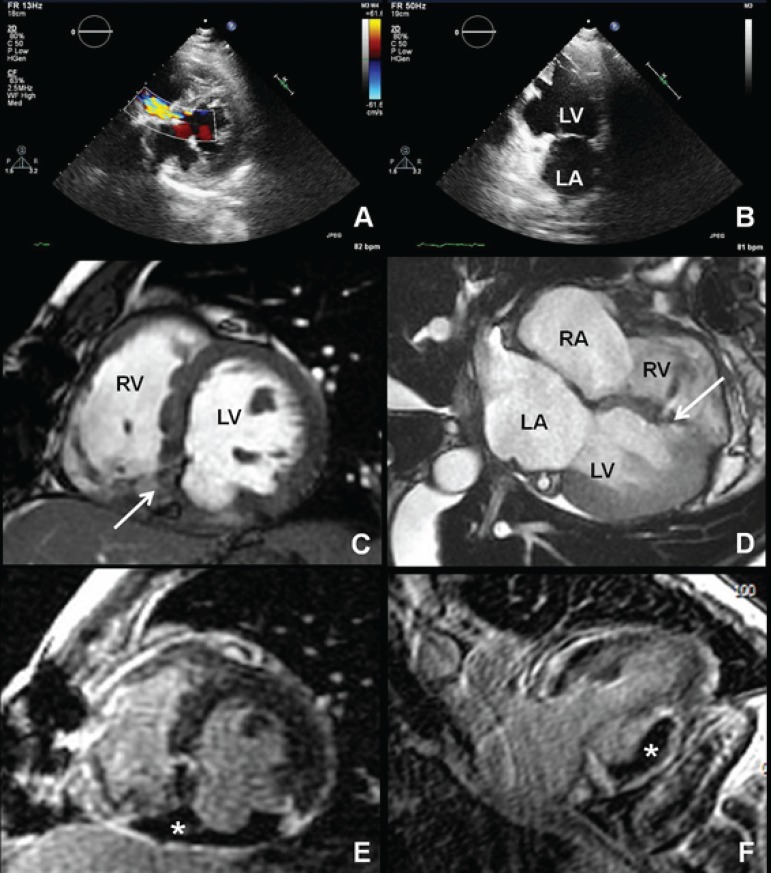
Echocardiographic mid-ventricular short axis view showing VSR with left-to-right shunt (A). Echocardiographic two-chamber view showing an inferobasal aneurysmatic region (B). Cardiac magnetic resonance (CMR) cine-imaging with a ventricular short axis (C) and four-chamber views (D) showing the accelerated flow through the ventricular septal defect (white arrow). CMR late-gadolinium enhanced images showing an inferobasal left ventricular pseudoaneurysm with a thrombus (star, E and F). RA: right atrium; LA: left atrium; RV: right ventricle; LV: left ventricle.

A 47-year-old male presented with a subacute inferior myocardial infarction (MI) complicated by inferobasal ventricular septal rupture (VSR) and underwent emergency surgical repair of VSR with a pericardial patch. In the postoperative period he remained hypotensive, with low exercise tolerance and a holosystolic murmur was present. Echocardiography showed persistence of VSR with left-right shunt (Figure 1A) and revealed an aneurysmatic region at the inferobasal wall of the left ventricle (LV), suggestive of a true aneurysm due to its large "neck" and smooth transition from normal myocardium to the aneurysm (Figures 1A and 1B). Cardiac magnetic resonance confirmed the VSR (Figures 1C and 1D) with significant shunt (Qp/Qs of 2.3), showed transmural necrosis of the basal and mid segments of the inferior and free wall of the right ventricle with moderate systolic dysfunction and also revealed that the aneurysmatic inferobasal region of LV was actually a pseudoaneurysm, without myocardial tissue but with a sessile and adherent thrombus within it (Figures 1E and 1F).

In conclusion, this patient presented with an inferior MI complicated by right ventricular infarction, inferobasal VSR and inferior pseudoaneurysm with organized thrombus inside. Mechanical complications are rare nowadays due to early revascularization. The multimodality imaging approach is essential for their correct diagnosis.

